# Pandem-ethics: Post-mortem reflection on healthcare worker ethics during the time of coronavirus disease 2019 pandemonium

**DOI:** 10.4102/sajid.v41i1.798

**Published:** 2026-07-31

**Authors:** Liesel H. Bösenberg, Veronica Ueckermann

**Affiliations:** 1 Department of Internal Medicine, Faculty of Health Sciences, University of Pretoria, Pretoria, South Africa; 2 Division of Infectious Diseases, Department of Internal Medicine, Faculty of Health Sciences, University of Pretoria, Pretoria, South Africa

**Keywords:** healthcare worker ethics, COVID-19 pandemic, moral theory, duty to care, social contract

## Abstract

**Background:**

The coronavirus disease 2019 (COVID-19) pandemic created ethical and legal tension for healthcare workers (HCWs), who had to balance professional obligations with personal safety, family responsibilities and resource limitations. The concept of ‘duty to care’ re-emerged as a contested ethical construct during periods of workforce vulnerability and healthcare scarcity.

**Aim:**

This article examines the ethical and juridical dimensions of HCWs’ duty of care during the COVID-19 pandemic, focusing on the South African constitutional framework, occupational health obligations and professional regulation, and whether this duty is absolute or subject to reasonable limits.

**Setting:**

The discussion is situated within South Africa’s healthcare response to the COVID-19 pandemic and the ethical expectations placed on clinicians in high-risk and emergency settings.

**Method:**

A narrative review was conducted using PubMed, Google Scholar and grey literature. Sources included peer-reviewed ethical analyses, legal frameworks and policy documents. A thematic synthesis analysed moral theory, legal duty, reciprocity and public health ethics.

**Results:**

The duty of care is substantial but not limited. South African law supports coexistence of rights rather than hierarchical obligations. Ethical analysis shows that proportionality, reciprocity and reasonable risk mitigation conditions professional duty. Employers must ensure adequate protection, transparent triage systems and psychosocial support. Failure to ensure reciprocity risks moral coercion and workforce attrition. The pandemic highlighted the need to define the thresholds where professional obligation yields to legitimate self-protection.

**Conclusion:**

The duty of care should be reconceptualised as a conditional, relational obligation grounded in constitutional values and public health ethics, with clear risk thresholds and enforceable reciprocal protections.

**Contribution:**

This article contributes to post-COVID-19 pandemic ethics literature by clarifying between the duty to treat, care and serve, proposing a Decatrad of operational reforms, reframing the duties as conditional, reciprocal and legally bounded.

## Introduction

The coronavirus disease 2019 (COVID-19) pandemic precipitated unprecedented strain on global healthcare systems, exposing healthcare workers (HCWs) to sustained clinical pressure, personal risk and moral uncertainty. Clinicians were required to navigate competing obligations: a professional duty to treat patients, a personal duty to preserve their own health and that of their families and a broader societal expectation to participate in the pandemic response.

In this context, the concept of a ‘duty to care’ re-emerged as a central yet contested ethical construct. While traditionally framed as a professional obligation grounded in beneficence and fiduciary responsibility, the pandemic revealed the limitations of this concept when applied in environments characterised by resource scarcity, inadequate protection and systemic inequity.

The South African context provided a particularly complex ethical landscape, shaped by constitutional rights, occupational health legislation, evolving societal guidelines and resource constraints. The coexistence of the right to emergency medical treatment and the right to a safe working environment illustrates the inherent tension between competing legal rights and HCW ethics.

The specific aims of this article are to examine the ethical and juridical foundations of the duty of care during pandemics and to interrogate whether this obligation is absolute or subject to limitation. It revisits the moral theory, professional codes and public ethics frameworks, and clarifies the judicial limits to care within the South African constitutional and labour law context. It also explores the role of reciprocity, the relevance of public health ethics frameworks and the need to redefine HCW, employer and state obligations in a manner that is both ethically defensible and operationally sustainable.

### A specific contribution to post-coronavirus disease 2019 pandemic ethics scholarship

A substantial body of scholarship published between 2020 and 2023 examined HCWs’ ethical obligations during the coronavirus disease 2019 (COVID-19) pandemic, with particular emphasis on the moral basis of the ‘duty to care’, professional risk-taking and the ethical justification for resource allocation during crisis conditions. Much of this literature emerged in real time as the pandemic unfolded, frequently emphasising normative ethical expectations grounded in beneficence, solidarity, altruism and professional virtue. While these contributions significantly advanced the discourse on pandemic ethics, important conceptual, juridical and operational gaps remain insufficiently addressed, particularly within resource-constrained health systems and jurisdictions characterised by strong competing-rights constitutional frameworks. This article contributes to the evolving post-pandemic discourse by addressing these gaps through a contextually grounded ethical and medico-legal analysis situated within the South African regulatory environment. International scholarship has extensively explored professional obligations during pandemics, yet relatively little work has systematically examined how the duty of care operates within South Africa’s constitutional architecture, where the right of access to healthcare services coexists with the right to a safe working environment. By analysing the interaction between Sections 24 and 27 of the Constitution, the *Occupational Health and Safety Act*, the *National Health Act* and professional regulatory guidance, this article demonstrates that HCW obligations exist within a framework of co-dependent rights rather than hierarchical ethical imperatives. In doing so, the article contributes a legally contextualised interpretation of professional duty, showing that the duty of care is neither absolute nor purely aspirational, but embedded within a reciprocal socio-legal contract between HCWs, institutions and society.

It specifically explains three related but frequently conflated constructs: the duty to treat, the duty to care and the duty to serve. Much of the pandemic ethics literature has used these terms interchangeably, contributing to ambiguity about the scope and limits of HCWs’ obligations in high-risk clinical conditions. This article distinguishes the duty to treat as a patient-centred obligation arising from the clinician-patient relationship and grounded in both legal and ethical principles relating to negligence and abandonment. The duty to care is presented as a broader professional responsibility to mitigate harm within healthcare systems, incorporating proportionality, competence and feasibility. The duty to serve is introduced as a population-level ethical concept that recognises the legitimacy of alternative contributions to pandemic response, including telemedicine, coordination, education, public health support and system-level functions. This conceptual clarification contributes to a more nuanced ethical vocabulary and supports more ethically defensible decision-making when frontline clinical care may expose HCWs to disproportionate or unjustifiable risk. The review aims to advance the literature by moving beyond descriptive ethical reflection towards operational ethical guidance. Early pandemic discourse often relied on appeals to altruism, solidarity and professional identity without sufficiently specifying the reciprocal responsibilities of institutions, employers and governments. Reciprocity is widely acknowledged in public health ethics frameworks as a key ethical principle underpinning legitimate expectations of professional duty. However, it has often remained an aspirational concept rather than an operationalised institutional obligation. Reciprocity is an ethically and legally enforceable requirement, emphasising that expectations of professional duty are contingent on the provision of adequate personal protective equipment (PPE), safe working environments, transparent triage policies and psychosocial support structures. By framing reciprocity as a necessary condition for ethical legitimacy, the review supports the shift from virtue-based appeals to a structurally grounded ethic of preparedness. In the post-pandemic reflective phase, 6 years after the onset of COVID-19, ethical analysis can move beyond reactive commentary towards prospective guidance. The pandemic served as a real-world stress test of ethical frameworks governing professional obligations, exposing persistent tensions between normative ethical expectations and operational realities characterised by resource scarcity, workforce vulnerability and systemic inequalities. Early narratives that valorised HCWs as ‘heroes’ have increasingly given way to recognition of burnout, moral injury and workforce attrition, highlighting the limitations of relying on moral exhortation alone to sustain healthcare systems during prolonged crises. This article contributes to the emerging shift from heroism-based narratives towards sustainability-based ethical frameworks that emphasise institutional accountability and shared responsibility. Translating ethical theory, legal precedent and public health ethics frameworks into a structured set of reform priorities aimed at strengthening preparedness for future public health emergencies, this review links ethical principles such as proportionality, reciprocity, solidarity, justice and transparency to institutional design and policy implementation, and supports the development of ethically defensible and operationally sustainable pandemic frameworks. A reactive ethical discourse is being transitioned towards proactive ethical preparedness, ensuring that future expectations of HCWs’ duties are clearly defined, contextually justified and supported by reciprocal institutional protections. This review offers a contextually grounded, conceptually clarified and practically oriented contribution to post-COVID-19 pandemic ethics scholarship, addressing persistent ambiguities regarding the scope and limits of the duty of care and supporting the development of ethically legitimate and sustainable HCW obligations in future public health emergencies.

## Methods

This study was conducted as a narrative review to critically synthesise ethical, legal and professional dimensions of HCW duty of care during pandemics, with the emphasis on the COVID-19 context and South African frameworks.

A structured, non-systematic research strategy was employed. Electronic databases, including PubMed and Google Scholar, were searched for peer-reviewed literature. In addition, grey literature sources were included to ensure contemporaneous relevance, including, but not limited to, policy documents, legal frameworks, professional regulatory guidance (e.g. Health Professions Council of South Africa [HPCSA], World Health Organization [WHO]) and government publications.

Search terms included combinations of the following keywords:

- ethics in pandemics
- duty to care
- duty to treat
- duty to serve
- ethical rights and obligations of HCWs
- occupational health and safety of HCWs
- refusal to work in healthcare ethics
- coronavirus disease 2019 HCW risks.

Boolean operators (ANDa/OR) were used to refine the results, and the reference lists of key articles were hand-searched to identify additional relevant sources.

Inclusion criteria:

- Peer-reviewed articles addressing ethical, legal or professional duties of HCWs during pandemics.
- South African legal, constitutional and occupational health frameworks.
- Internal ethical guidelines and public health ethics frameworks.
- Articles published in English.

Exclusion criteria:

- Studies or articles unrelated to HCW roles in pandemic contexts.
- Purely clinical studies without ethical relevance.

Sources were thematically synthesised rather than quantitatively analysed, consistent with narrative review methodology. The analysis focused on key domains: moral theory, legal obligations, public health ethics, reciprocity and professional regulation.

### Interesting historical perspectives

The plague’s impact during the Middle Ages was devastating, reducing the European population by half by 1400. The 1918–1919 Spanish flu pandemic occurred in three waves and is estimated to have killed between 20 million and 100 million people in just 1 year. It spread across every continent except Antarctica.^1,2^ The deadliest disease in recorded history wiped out 8% – 10% of people aged 20–40 years. Around 600 civilian doctors in the United States (US) and 2% of South African physicians died from the Spanish flu.^3^ Pandemics also occurred in 1957, 1968 and most recently in 2009 with the Influenza A (H1N1) pandemic. Throughout history, doctors have often refused to see patients during plagues. Even the renowned physician Galen fled Rome during the Antonine Plague in 165–180 AD, and in 1382, Venice passed laws forbidding doctors from abandoning their duties.^4^ These historical anecdotes show that tensions between professional duty, personal risk, and societal expectation are not unique to the recent COVID-19 pandemic but are an enduring aspect of pandemic medicine, highlighting the need for a clearer operational definition of HCWs’ ethical responsibilities in contemporary practice.

### Societal guidelines and standards for ethical behaviour

Dr Sandeep Jauhar, a cardiologist, wrote a persuasive article in the *New York Times* in April 2020. He highlighted that the American Medical Association (AMA), in its first Code of Medical Ethics in 1847, stated that ‘when pestilence prevails, the physician must face the danger and continue their labours for the alleviation of suffering, even at the jeopardy of their own lives’. Clark, in his article ‘*In Harm*’s Way: AMA physicians and the duty to treat’, states that there has been a longstanding tension between physicians’ autonomy rights and societal obligation. It appears that there has been a longstanding undercurrent of dissatisfaction with the absolute, unflinching enforcement of the ‘duty to care’ in ethical codes.^5^ In Chapter 8, opinion 8.3 of the current AMA Code of Ethics (2004), ‘commitment to care for sick and injured’ requires physicians to perform urgent medical care during disasters. This ethical responsibility remains even in the face of greater-than-usual risks to the physician’s safety, health or life, and in the most recent version of this living ethics document, it is stated that:

At the national, regional, and local levels, responses to disasters require extensive involvement from physicians, both individually and collectively. Because of their commitment to care for the sick and injured, individual physicians have an obligation to provide urgent medical care during disasters. This obligation holds even in the face of greater-than-usual risks to physicians’ own safety, health, or life. However, the physician workforce is not an unlimited resource. Therefore, when providing care in a disaster with its innate dangers, physicians also have an obligation to evaluate the risks of providing care to the individual patient versus the need to be available to provide care in the future.^6,7,8^

In the 2022 update of the World Medical Association Code of Ethics, it states that ‘the physician should provide help in medical emergencies, while considering the physician’s own safety and competence, and the availability of other viable options for care’.^9^

At the start of the pandemic, the Ethical Guidelines and the Code of Conduct (Booklets 1 & 2) of the HPCSA outlined practitioners’ general duties. However, they did not specify tasks during pandemic situations or natural disasters. In May 2020, the HPCSA shared links to the National Institute for Communicable Diseases (NICD) COVID-19 Practical guidelines for HPCSA practitioners, which offer advice on the practical management of COVID-19 in clinical settings. It is essential to recognise, however, that these booklets function within a broader South African legal framework and are not standalone.

As professional codes evolve, they reflect the tension between aspirational ethical commitments and the practical realities of delivering care amid situations with heightened risk.

### Waiting for the tsunami to hit

As South Africa anticipated the first wave of COVID-19 infections, HCWs faced competing professional, personal and societal obligations amid uncertainty, heightened occupational risk and resource constraints. Ethical debate focused on whether entry into the healthcare professions implies implicit consent to assume heightened personal risk during public health emergencies, and whether societal expectations of professional selflessness may justifiably limit HCWs’ rights to personal safety and acceptable working conditions. The extent to which professional commitment entails an obligation to provide care in environments where PPE or institutional preparedness may be inadequate became a central point of ethical and legal scrutiny. These tensions highlighted the importance of reciprocity, emphasising that expectations of professional duty are contingent upon corresponding obligations of employers, professional bodies and the state to ensure reasonable risk mitigation, transparent governance and equitable allocation of resources.

Vulnerable subgroups of HCWs (e.g. those close to retirement age or living with co-morbidities) faced significant risks of infection. Employers had a legal obligation under human rights law to accommodate workers with particular vulnerabilities without discrimination and to ensure a safe working environment. Unfortunately, refusing to work or even striking under unsafe conditions would not fully protect against possible negligence and other liability claims if a breach of the legal duty of care were proven. However, HCWs could not act entirely autonomously, nor could it be assumed that they must work under any circumstances during a pandemic.^10,11,12^

According to the WHO document ‘The Role and Obligations of Healthcare Workers during an outbreak of pandemic influenza’, human resources are essential to an effective pandemic response. The ethical core values ought to be balanced with changes in employment contracts to include flexible hours, access to a safe working environment, special leave arrangements and pandemic duty pay packages to ensure the effectiveness of any national pandemic preparedness plan. In the US, this pandemic preparedness plan stated that employees could refuse to work only under very specific circumstances, and even then, they risked disciplinary action:

- If the employee believes in good faith that the work would put them in serious and immediate danger.
- If they have asked the employer to remedy the hazard, with no response.
- If there is no other way to perform the task safely.

### Legislative frameworks regulating healthcare worker duties in South Africa

These ethical tensions are mirrored and, at times, intensified within the legal frameworks that govern healthcare practice in South Africa. Although pre-existing juridical frameworks were in place at the start of the pandemic, their practical operationalisation during its swift escalation generated significant uncertainty at the clinical coalface. Much had been written concerning the ethical duties of HCWs to provide treatment during pandemics, but very little about their legal responsibilities. A breach in the legal duty of care or patient abandonment may give rise to a claim in negligence. If a patient–doctor relationship exists, abruptly ending it could result in a negligence lawsuit if the patient is harmed or abandoned, especially if such liability was foreseeable. Emergency care cannot be refused (Section 27[3]) of the *Constitution of the Republic of South Africa, 1996*: ‘No one may be denied emergency medical treatment’. Chapter 2(5) of the *National Health Act 61 of 2003* states: ‘A health care provider, worker or health establishment may not deny a person emergency medical treatment’. However, this must be balanced with medical workers’ rights to work in conditions that do not pose unacceptable health and safety risks.^13,14^

In his article, ‘COVID-19: what should employers do if employed health professionals such as doctors and nurses refuse to treat COVID-19 patients despite being provided with the required personal protective equipment?’, McQuoid-Mason^15^ gives clear guidance on how the refusal to work is viewed within the South African legal framework. In Section 24 of the Constitution, everyone has the right to an environment that is not harmful to health or well-being, and the *Occupational Health and Safety Act 85 of 1993* (OHSA), places a statutory duty on employers to provide and maintain a working environment that is safe and without risk to the employee’s health, including the provision of PPE. Under the OHSA, employees may refuse to work only where there is imminent and serious danger that the employer has failed to address. The *Labour Relations Act 66 of 1995* does not protect refusal to work where adequate PPE and reasonable safety measures are in place, and such refusal may constitute misconduct. This legal position is reaffirmed by the HPCSA ethical rules, which oblige practitioners to place patient interests first, while recognising that this duty is contingent on employers fulfilling their reciprocal legal obligation to protect staff.^15^

In March 2020, the South African Department of Employment and Labour published a guideline document, *Workplace Preparedness: COVID-19*, that introduced risk categories and linked employer duties under the OHSA.^14,16^ The Critical Care Society of South Africa published a document on the allocation of scarce critical care resources in April 2020. This document grounded intensive care unit (ICU) triage during a public health emergency in core ethical principles: duty to care, stewardship of scarce resources, distributive and procedural justice and transparency, shifting the ethical focus from individual patient benefit to maximising population-level outcomes. It justified prioritisation, reassessment and even withdrawal of critical care using objective criteria to guarantee fair, consistent and morally defensible decisions while protecting dignity, palliative care and due process for those who cannot receive or continue ICU treatment.^14,17,18^ The South African Medical Association also published an ethical guide to managing COVID-19-related ethical issues during this time.^19^

The ethical framework expressly for the severe acute respiratory syndrome coronavirus 2 (SARS-CoV-2) pandemic was largely uncharted territory, with many competing but coexisting rights. Some pre-existing regulatory structures provided guidance to organisations and healthcare practitioners. The first of these is South Africa’s Constitution, which provides an ethical-legal framework for pandemic decision-making. Section 24 of the Constitution guarantees the right to an environment that is not harmful to health or well-being. This includes a safe working environment, which is directly applicable to HCWs exposed to infectious risks. During the pandemic, shortages of PPE, long working hours and staff shortages threatened this right. Section 27 of the Constitution, which affirms the right to access healthcare services and explicitly prohibits the refusal of emergency medical treatment, illustrates a competing-rights framework. These rights were not absolute and co-existed in tension during the pandemic – in fact, they appeared to conflict when healthcare resources were overwhelmed by the number of sick patients. The *National Health Act 61 of 2003* operationalises constitutional rights and responsibilities. The refusal of medical treatment by healthcare providers and facilities was prohibited in light of the legal duty of care owed to patients. The act does not define the limits of such duties for clinicians and institutions and, at times, appears to contradict the rights of overworked and under-resourced healthcare personnel, as recognised in section 20 of the same act. Explicit legal duties of healthcare employers are set out in the OHSA, which clarifies that HCWs’ obligation to treat is conditional on reasonable risk mitigation, rather than an open-ended duty to accept preventable harm. The OHS framework highlights the employer’s obligation to provide a safe and healthy working environment, implement symptom screening and referral systems, provide training in infection prevention and control (IPC) and provide adequate and appropriate PPE. In 2022, the International Labour Organization, at the International Labour Conference, also recognised safe and healthy working conditions as its fifth fundamental principle, ethically requiring employers, workers and governments to actively protect life, health and well-being in all working environments.^14^

Under the *Disaster Management Act*, COVID-19-specific occupational health regulations were promulgated. These included mandatory workplace IPC measures, risk-stratified worker management and enforcement of compliance with OHS standards. The act translated ethical principles into enforceable public health obligations during the ‘State of disaster’ declared in South Africa during the pandemic. The *Compensation for Occupational Injuries and Diseases Act* provided compensation for HCWs who contracted SARS-CoV-2 through workplace exposure.^20^

In May 2020, the national and departmental plans clarified the rights and responsibilities of stakeholders during the pandemic. The National Plan for COVID-19 Health Response of May 2020 classified HCWs as a high-risk group and mandated the provision of adequate PPE and the reporting of PPE shortages. It also addressed mental wellness support, rest and surveillance for HCWs.^21^ In July 2020, a specific COVID-19 workplace exposure directive clarified the process and eligibility criteria. This directive provided a post-exposure safeguard that supported the societal benefit derived from the work of healthcare providers, who have reciprocal obligations of restitution. By August 2020, the ‘Strategy to protect the health and safety of health workers’ provided a framework for the physical, psychological and organisational protection of health workers. These national policies demonstrated that HCWs were not operating in a regulatory vacuum and that their rights were not nullified by the duty of care.^22^

South Africa’s labour framework is grounded in a global ethical consensus that recognises OHS as a fundamental right in the workplace. South Africa had an early and relatively sound regulatory framework, with shared responsibility shared by the state, employers and clinicians. The framework recognised the vulnerability of HCWs and aimed to mitigate risk and compensate for harm. The frameworks were designed to address uncertainty at the clinical ‘coalface’ of care provision under extremely challenging circumstances.

### Ethical values at the core of pandemic planning

Beyond legal obligations, pandemic response is fundamentally shaped by underlying ethical values that guide both individual and institutional decision-making. It is generally accepted that HCWs have an ethical and legal duty to treat patients. The dilemma arises when the noble sentiment of a role-based obligation to save patients conflicts with the HCW’s right to preserve their own life and health. The right to life is enshrined in the South African Constitution (1996), Chapter 2(11), and, by extension, the right to a safe working environment, as stated in the *National Health Act 61 of 2003* (Chapter 2(20): The rights of healthcare personnel). The only way to overcome this gap is to address the physical working environment needs of staff and functions, and to implement ethical values relevant to pandemic planning.

Ten core ethical values underpin pandemic planning.^23^ As far as *individual liberty* is concerned, *the protection of society* as a whole will take priority. Employers, institutions and governments must implement measures that balance individual liberty with necessary protections, ensuring they are proportional to the threat (the principle of *proportionality*). The *privacy* and *confidentiality* of individual patients remain sacrosanct, but may be overridden when protecting others becomes paramount. Healthcare workers are also human and share patients’ fears and concerns. That is why their obligation to adhere to a code of ethics directly conflicts with the *duty to care*. Healthcare workers also need care and support to balance their responsibilities with their personal lives. However, the burden of combating the pandemic is not borne solely by the healthcare fraternity. *Reciprocity* is a concept whereby society acknowledges the burden on HCWs and supports them by complying with lockdown rules, such as practising social distancing, washing hands and wearing masks. *Equity* overlaps with the well-known ethical principle of distributive justice, ‘wherein the care provided to patients should be done equitably’. That is certainly the biggest challenge healthcare workers face, especially in resource-constrained settings. For a pandemic plan to work well and succeed, a high level of *Trust* must exist between health workers, patients and administrators. Honest and explicit communication among all parties is fundamental. Decisions concerning resource *Stewardship* and treatment criteria should be a simple, universal collaboration among all stakeholders, promoting solidarity and clear leadership in establishing a strategic pandemic plan.^23^

## The concept of ‘duty to treat’: The moral theory

We all share a common morality, which reflects our common humanity. Generally, as human beings, we accept a moral duty to help others in need while minimising risk to ourselves, such as administering the Heimlich manoeuvre to someone who is choking. In the moral theory, this is referred to as a ‘general positive duty’ (also known as beneficence). The concept of a ‘special positive duty’ refers to a duty that requires a person to assume a greater personal risk, such as HCWs caring for patients during a pandemic. The dilemma arises when a qualified HCW questions the ‘special positive duty’: should they leave the profession if no alternatives are provided?

The so-called ‘duty to treat’ generally rests on two principles: the moral obligation and the legal duty to provide care. Sokol^24^ states that this ‘nebulous’ term might become an instrument of intimidation, pressuring HCWs to work in circumstances they consider morally, psychologically or physically unacceptable. He argues that this phrase might be ‘ethically dangerous’ by generating the illusion of a legitimate moral justification. Healthcare workers have long recognised that the personal risks of caring for patients may be too high and that it may be unethical to refuse. Many are also concerned about the personal liability and negligence risks associated with such a refusal. Additionally, if medical professionals were to disclaim their duty of care during a pandemic, would their professional reputation suffer from making such a choice? Proponents of the moral obligation argue that HCWs should fulfil their ethical duties regardless of the circumstances. However, no HCW has ever explicitly consented to work during a pandemic, except perhaps in cases where employment is contractually agreed upon and explicitly states so. Therefore, the question arises: Does the fact that newly qualified healthcare professionals never explicitly oppose pandemic care imply their consent? Can we assume that HCWs have truly consented to the intrinsic risks of their profession simply because these dangers are standard and they failed to object initially? Should physicians who accept some level of risk be exposed to dangers that nearly amount to ‘suicide’?

The main points and philosophical arguments of the moral theory, urging medical professionals to honour their duty to treat during pandemics, are the following:

- A professional career in medicine came with an explicit or implicit acceptance of such risks when the individuals entered into it (so-called *implied consent*).
- It is implicitly part of the code of ethics that healthcare workers undertake when they enter the profession.
- A higher burden of responsibility is placed on healthcare workers due to their *special training* and expertise.
- Because the health professionals received benefits of subsidised training, have the privilege of a higher social status, perceived higher income and self-regulative autonomy, a *Social contract* now exists with the public that needs to be honoured.^25^

This view of reciprocity acts as a strict taskmaster. It insists that HCWs, in exchange for the benefits of a self-regulated profession, have a duty to treat patients, even if this entails greater-than-normal risk to themselves. As a result of their professional obligation, HCWs are expected to put themselves in harm’s way and bear a disproportionate share of the personal risk during a pandemic.^26^ If individual HCWs refuse, they are seen as ‘free-riders’, meaning they benefit from being part of the healthcare profession without fulfilling their fair share of the work.^25^ In his book about the Great Plague in London of 1665, *A Journal of the Plague Year*, Daniel Defoe notes that most physicians who did not participate in patient care were called ‘deserters’, which points out the societal bias against HCWs if they choose not to participate due to personal safety or ethical concerns.^27^ Reciprocal obligations by employers, society, and government should aim to preserve the worker’s ability to care for patients and to recognise the added risks that they are assuming.^26^

### Conceptual clarification between the ‘duty to care’, the ‘duty to treat’, versus the ‘duty to serve’

As mentioned earlier in the article, the ethical ambiguity surrounding HCW obligations during pandemics is partly attributable to the interrelated terms of duty to treat, duty of care and duty to serve. These concepts represent distinct ethical constructs with different implications for ethical obligation and acceptable risk.

#### Duty to treat

The duty to treat refers to a direct, patient-centred obligation arising from the clinician-patient relationship. It is both ethical and legal, where the ethical code is grounded in beneficence and professionalism, and the legal aspect, where abandonment of patients or refusal may constitute negligence.

#### Duty to care

This is a broader and more context-dependent professional obligation, encompassing the responsibility to mitigate harm and contribute meaningfully to patient and system-level outcomes. Unlike the duty to treat, it extends beyond individual patient encounters. It incorporates professional judgement regarding risk, competence and resource allocation and allows for conditional engagement, particularly in high-risk environments. It is best understood as a qualified ethical obligation, shaped by proportionality, reciprocity and feasibility.

#### Duty to serve

The duty to serve represents a population-level and systems-oriented obligation, emphasising contribution to the broader pandemic response rather than direct clinical care. This may include HCWs taking on public health roles, administrative or coordination functions, telemedicine and remote care, and training and support roles. The duty to serve provides an ethically defensible alternative where frontline clinical care is not feasible due to personal risk, vulnerability or competing obligations.

The healthcare professional is subject to several competing duties: a duty to patients, a duty to protect themselves from undue risk and harm, a duty to their family, a duty to colleagues whose workload and risk of harm will increase in their absence and, lastly, a duty to society.^7,25^ Malm et al.,^28^ in the sentinel article ‘Ethics, pandemics, and the duty to treat’, indicated that the classical arguments that aim to persuade healthcare workers to adhere to a ‘duty to treat’ can be disclaimed, especially if the healthcare workers consider their competing responsibilities to themselves and families more important.

Without breaching clinical responsibility, the ‘duty of care’ could be then defined as ‘a responsibility to pursue a variety of ends to mitigate the negative effects of a pandemic’, which could open opportunities for healthcare workers to commit to and support a pandemic response in different ways, the so-called ‘duty to serve’.^23,25^ This implies that the emphasis is shifted from the traditional doctor–patient relationship (which is the main focus in the notion of ‘duty to treat’ and ‘duty to care’) to that of serving the greater public during the pandemic for the greater good.^23,24,25,29,30^

### The limit to the ‘duty of care’ and acceptable risk levels

In the article ‘Lives on the line? Ethics and practicalities of duty of care in pandemics and disasters’, the authors argue that a physician’s duty of care is not unlimited. It is shaped by several factors, including the physician’s primary speciality, the burdens and risks the clinician faces, the potential benefit of treatment to patients, and the clinician’s multiple roles in their personal life.^6^ They, as so-called ‘multiple agents’, have two spheres of obligation, professional and personal, which are separate and at the same time overlapping.^24^ It is during times of crisis that the multiple roles HCWs fulfil come into conflict.

Defining acceptable risk levels will largely depend on the HCW’s area of expertise. Again, the argument is made that by working in a specific speciality, for example, emergency medicine, infectious diseases or critical care, they implicitly consent to the risks and responsibilities associated with their job. When the risk ceases to be acceptable, and who makes that decision, are questions open to debate. If a HCW opts out of direct patient care, the request should be assessed on an individual basis, considering compelling personal circumstances, such as advanced age, immunocompromised status or single parenthood. The worker should be given the option to contribute in a non-clinical way, recognising that they might, as a result, suffer from ‘side-line guilt’.^22,29^

‘Duty to care’ is a term that Daniel Sokol already, in 2006, referred to as ‘ethically dangerous’, because the nature and the scope of the duty require definition. He argued that conflicting duties should be recognised and acknowledged, as they are not fixed or absolute and are heavily context-dependent.^31^ He believed this ethical phrase could guilt HCWs into working in unacceptable, risky environments while upholding an ‘illusion of legitimate moral justification’.^24^ In the aftermath of COVID-19, many argue that the ‘duty to care’ and ethical obligation arguments have become outdated, ambiguous and vague. A new dialogue is needed to address the issues surrounding this ethical concept and to determine how explicitly and rigorously this duty should be defined and applied in practice.^32^ The duty to care should be reframed not as a moral absolute, but as a bounded, context-sensitive obligation embedded within a reciprocal socio-legal contract.

The ethical discourse surrounding the duty to care must move beyond the binary interpretations of obligation versus abandonment. The pandemic demonstrated that unqualified appeals to professional duty risk functioning as instruments of moral coercion, particularly in under-resourced systems. Ethical legitimacy requires that the duty is conditional, reciprocal and operationally defined, rather than rhetorically invoked. Without enforceable protections, appeals to altruism exploit professional identity while externalising systemic failure onto individual HCWs.

### Virtuous workers, patients and employers: Mutual tolerance and compassion

Healthcare worker ethics must be understood within the wider moral failures of global political and economic systems rather than reduced to individual conduct alone. Benatar argues that COVID-19 exposed deep structural injustice, where workers were expected to absorb the risk, scarcity and moral distress while remaining compliant within inequitable systems – an ethically untenable form of moral docility. Applying his relational and solidarity-based ethic, HCWs not only have duties of care to individual patients but also a collective ethical responsibility to resist injustices, advocate for fair resource allocation and speak out against policies that entrench global and local inequities. Mutual caring, in this framing, requires moral courage and professional solidarity, positioning HCWs as ethical agents who challenge unjust structures rather than passive participants in their reproduction.^33^

Dhai’s articulation of care and altruism in medical ethics grounds compassionate care in virtue ethics, where moral character, empathy and moral imagination guide ethical action beyond rigid, rule-based obligations. Altruism in healthcare is not self-sacrifice without limits. It represents a moral practice sustained via mutual respect and tolerance among HCWs, patients and employers. Virtuous HCWs demonstrate compassion through integrity; patients participate as moral agents through trust, reciprocity and respect; and employers bear ethical responsibility to create enabling, just and supportive environments. Together, these form a moral community in which compassion is shared rather than exploited, encouraging mutual tolerance and ethical resilience, chiefly in contexts of crises and constraint.^34^

### Nursing, vulnerability and moral agency in pandemic ethics

Nurses occupy a uniquely vulnerable and ethically complex position within pandemic healthcare systems, functioning at the interface of prolonged patient contact, institutional hierarchy and often limited decision-making power. The work of Mulaudzi et al.^11^ provides critical insights into this tension, describing nurses as being ‘caught between a rock and a hard place’, where professional expectations collide with legitimate concerns for personal safety and labour rights.

Unlike physicians, whose ethical discourse often centres on autonomy and professional duty, nursing ethics is deeply rooted in relational care, proximity and sustained patient engagement, which amplifies both moral burden and physical risk. Mulaudzi highlights that during COVID-19, nurses frequently experienced inadequate PPE provision, limited institutional support, suppression of collective protest or refusal to work and heightened exposure due to bedside care roles.

This creates a significant ethical dilemma: while nursing professionalism emphasises compassion and continuous care, structural inequalities limit nurses’ capacity to exercise moral agency. Importantly, Mulaudzi reinterprets refusal to work not as abandonment but as a legitimate ethical response to unsafe conditions, based on labour rights and occupational justice. This reflects a broader shift towards recognising HCWs not just as duty-bearers but as rights-holders.

Pandemic ethics must therefore explicitly include nursing perspectives, recognising the disproportionate burden on nurses, the ethical legitimacy of protest when conducted safely and the need for institutional accountability. Failing to do so risks entrenching hierarchical ethical frameworks that valorise sacrifice while concealing systemic injustice.

### Public health ethics frameworks

In a brief commentary, Lundgren and Etheredge describe public health ethics frameworks as structured approaches that balance public well-being alongside individual rights and emphasise proportionality, necessity, least infringement and transparency in public health decision-making. These frameworks recognise that public health interventions are inherently normative and require explicit ethical justification, particularly when resource-allocation measures restrict autonomy or impose burdens on specific groups. By stressing accountability, equity, transparency and public justification, ethical analysis guides real-world governance and population-level risk management, guaranteeing that public health action remains morally defensible, particularly during emergencies such as the COVID-19 pandemic.^35^

## Review findings

### Post-pandemic reflection

Six years after the onset of the COVID-19 pandemic, the ethical questions surrounding HCWs’ duty of care remain unresolved, but no longer abstract. The pandemic functioned as a real-world stress test of ethical frameworks, exposing critical gaps between normative ethical expectations and operational realities. Several enduring lessons underscore the continued relevance of this ethical discourse:

#### Persistence of systemic vulnerabilities

Resource constraints, workforce shortages and inequitable healthcare infrastructure remain defining features of many health systems, particularly in low- and middle-income settings. The conditions that generated ethical conflict during COVID-19 have not been fundamentally resolved.

#### Shift from heroism to sustainability

Early narratives of HCWs as ‘heroes’ have given way to recognition of burnout, moral injury and workforce attrition, necessitating a transition from valorisation to sustainability-focused ethics.

#### Emergence of legal and policy precedents

Coronavirus disease 2019 established practical precedents for triage, occupational risk thresholds and refusal to work. These now require formalisation into enduring policy rather than remaining crisis-specific responses.

#### Heightened emphasis on reciprocity

The failure of systems to consistently uphold reciprocal obligations (e.g. PPE provision, psychosocial support) has reinforced the view that duty without reciprocity is ethically indefensible.

#### Preparedness for future pandemics

With increasing likelihood of future pandemics, ethical clarity is no longer theoretical – it is a preparedness imperative. Thus, the topic remains highly relevant as it contributes to the transition from reactive ethical discourse to proactive, structured pandemic ethics frameworks.

### A ‘Decatrad’ of recommendations for ethical, legal and institutional reform

Having clarified the conceptual distinctions between the different ethical duties, as well as the juridical limits of professional obligation within the South African constitutional framework, the proposed recommendations for reform can be grouped into 10 main focus areas, those being:

- **The definition of explicit risk thresholds:** Regulatory bodies, for example, the HPCSA, should formally define acceptable occupational risk thresholds, including criteria for a legally justified refusal to work.
- **The development of pandemic-specific ethical guidelines:** Ethical codes must include context-specific provisions for pandemics that address triage, duty limits and role flexibility.
- **Strengthening of reciprocity mechanisms:** Governments and institutions must operationalise reciprocity through a guaranteed PPE supply chain, hazard compensation, mental health support and occupational injury compensation.
- **Integrating legal literacy into healthcare worker training:** A fresh look should be taken at incorporating legal literacy into undergraduate and postgraduate curricula, with a focus on constitutional rights (Sections 24 and 27), the provisions of the *Occupational Health and Safety Act* and the legal implications of negligence and patient abandonment.
- **Formalise alternative roles (‘duty to serve’):** Healthcare workers unable to perform frontline duties should be redeployed into telemedicine, public health coordination, training and supervisory roles.
- **Mobilising institutional support for ethical deliberation:** There is still a dire need for institutional clinical ethics committees that are accessible during crises to support real-time decision-making.
- **Recognise and protect the workforce:** Policies must explicitly address doctor- and nurse-specific tasks and protect the right to protest unsafe conditions for both staff and patients. Equitable allocation of resources should be ensured at all times.
- **Embedding public health ethics frameworks into policy:** Decision-making should constantly reflect the principles of proportionality, transparency, equity and accountability.
- **The implementation of monitoring and accountability systems:** Oversight mechanisms should function independently and evaluate workplace safety compliance, institutional ethical conduct and the implementation of pandemic policies.
- **Re-shaping of global and national solidarity frameworks:** Ethical preparedness must extend beyond institutional or country-specific borders and lead to a global, cohesive and unified framework that embraces public compliance, reciprocity measures and international cooperation in equitable resource allocation.

The ‘Decatrad’ represents not merely aspirational ethical guidance, but a structured ethical and practical approach to align normative principles with implementable safeguards for HCWs and patients.

## Conclusion

The COVID-19 pandemic exposed the fragility of traditional ethical constructs that underpinned HCW obligations. It posed a challenge for HCWs. Frontline clinicians were left oscillating between professional duty and existential vulnerability. It became clear that the healthcare profession had moved beyond the days of unquestioned ‘noble sacrifice’ and the moral coercion of ‘duty to treat’. The notion of unconditional duty of care is neither ethically defensible nor operationally sustainable in the absence of reciprocal protections and clearly defined limits.

The post-pandemic moment demands a move away from rhetorical invocations of ‘duty’ to a clearer articulation of risk thresholds, enforceable reciprocal protection, structured ethical deliberation and legally coherent regulatory structures. Clarifying the boundaries of the duty to care does not undermine professional ethics; it is essential to uphold ethical legitimacy, workforce resilience and public trust during times of collective vulnerability.

Without such reform, the ethical burden of future pandemics risks being borne once again disproportionately by individual HCWs, rather than being equitably distributed across the systems and societies they serve.
